# Evaluation of Construct and Criterion Validity for the ‘Liverpool Osteoarthritis in Dogs’ (LOAD) Clinical Metrology Instrument and Comparison to Two Other Instruments

**DOI:** 10.1371/journal.pone.0058125

**Published:** 2013-03-07

**Authors:** Myles Benjamin Walton, Emily Cowderoy, Duncan Lascelles, John F. Innes

**Affiliations:** 1 Department of Musculoskeletal Biology and School of Veterinary Science, University of Liverpool, Neston, Wirral, United Kingdom; 2 Comparative Pain Research Laboratory and Center for Comparative Medicine and Translational Research, and College of Veterinary Medicine, North Carolina State University, Raleigh, North Carolina, United States of America; University of Sydney, United States of America

## Abstract

**Objective:**

To test the ‘Liverpool Osteoarthritis in Dogs’ (LOAD) questionnaire for construct and criterion validity, and to similarly test the Helsinki Chronic Pain Index (HCPI) and the Canine Brief Pain Inventory (CBPI).

**Design:**

Prospective Study.

**Animals:**

222 dogs with osteoarthritis.

**Procedure:**

Osteoarthritis was diagnosed in a cohort of dogs on the basis of clinical history and orthopedic examination. Force-platform analysis was performed and a “symmetry index” for peak vertical force (PVF) was calculated. Owners completed LOAD, CBPI and HCPI instruments. As a test of construct validity, inter-instrument correlations were calculated. As a test of criterion validity, the correlations between instrument scores and PVF symmetry scores were calculated. Additionally, internal consistency of all instruments was calculated and compared to those previously reported. Factor analysis is reported for the first time for LOAD, and is compared to that previously reported for CBPI and HCPI.

**Results:**

Significant moderate correlations were found between all instruments, implying construct validity for all instruments. Significant weak correlations were found between LOAD scores and PVF symmetry index, and between CBPI scores and PVF symmetry index.

**Conclusion and Clinical Relevance:**

LOAD is an owner-completed clinical metrology instrument that can be recommended for the measurement of canine osteoarthritis. It is convenient to use, validated and, as demonstrated here for the first time, has a correlation with force-platform data.

## Introduction

Osteoarthritis (OA) is estimated to affect approximately 20% of the adult dog population [1] causing reduced mobility, behavior changes [2], and altered activity patterns [3]. With an estimated canine population of 72 million in the USA (American Pet Products Association, 2011–2012 National Pet Owners Survey), this estimate indicates that over 14 million dogs are affected by OA. Such a common disease, in a species that gleans much of its quality of life from physical activity, has significant animal welfare implications. Furthermore, in 2005 the annual cost of the treatment of dogs with NSAIDs in the USA alone was estimated to exceed US$130 million, and at that stage was growing by approximately 13 per cent a year [4]. For a disease of such importance, it is essential that veterinarians can stage the disease for a particular animal. This is important for not only choosing appropriate treatment, but also monitoring disease progression, and for measuring the efficacy of treatments.

A “construct” is a term borrowed from clinical psychology and refers to any theoretical framework. The construct of OA is complex: that is, the clinical picture of an affected dog will include changes in limb function, ability to perform activities, overall activity, and demeanor, amongst other dimensions. Measurement of ground reaction forces (GRFs) using a force platform, or a pressure sensitive walkway, is generally considered the ‘gold standard’ for quantifying canine limb function [5], [6], [7], [8]. More recently, the potential usefulness of body-mounted, accelerometer-based activity monitors (AMs) in dogs with OA has been demonstrated [3], [9], [10], [11], [12], [13]. These are both objective, external measures that may capture differing dimensions of the OA construct.

In the field of human medicine, the standard methods for assessing chronic pain are validated, patient-completed, clinical metrology instruments (CMIs) [14]. A CMI is a sequence of questions or “items” that are scored based on the observations or experiences of the person completing it. These individual item-scores are then used to calculate an overall instrument score.

The design and testing of metrology instruments is well described [15], [16], [17]. The clinical usefulness of an instrument depends on its ease of use, which is influenced by its readability and the choice of scale, and also on its validity. It is important to note that validity is not a dichotomous variable, but a continuous one. The more evidence that can be provided for an instrument, the more “valid” it becomes, until a point when further validation adds little to the evidence base [15], [16].

Validity is sub-divided in to four major categories. *Face validity* answers the question “Is this instrument measuring what it is designed to measure?” and is usually tested through review by experts in the field, and/or review by the intended audience. *Content validity* answers the question “Is there anything missing that would add value?” and is also tested via a review process. *Construct validity* is a test of how well the instrument’s authors’ theorized construct matches the true construct of what is being measured. It can be tested by administering the instrument to groups of known and differing clinical status (e.g. those with OA and those without); by comparing the results of the instrument with those of other, similar measures; and by factor analysis. *Criterion validity* is a test of how well an instrument correlates with a standard, external measure of the disease. The validity of an instrument also depends on its *reliability* and its *responsiveness*. Reliability is a test of how well the instrument returns the same score for a given level of disease. It is also sub-divided into *repeatability* and *internal consistency*. Repeatability is examined in a test-retest scenario, over a period when it is assumed there is no change in the underlying level of disease [18]. Internal consistency is most frequently tested using Cronbach’s alpha [19].

There are at least six CMIs reported for measuring the severity of OA in dogs [17], [20], [21], [22], [23]. This report further investigates the ‘Liverpool Osteoarthritis in Dogs’ (LOAD) instrument, and, for the first time, compares it with the Canine Brief Pain Inventory (CBPI) and the Helsinki Chronic Pain Index (HCPI). Table 1 summarizes the psychometric testing of these instruments, as published in peer-reviewed literature at the time of writing.

**Table 1:** Published psychometric testing of LOAD, CBPI and HCPI.

INSTRUMENT	Face/Content Validity	Construct Validity	Criterion Validity	Reliability – Internal Consistency	Reliability - Repeatability	Responsive-ness
HCPI (in Finnish)	Y [30]	Y (Extreme groups[30], PFA [23])	Not Tested	Y [23]	Y [23]	Y [23]
CBPI	Y [29	Y (PFA, QOL question [29])	Not Tested	Y [29]	Y [29]	Y [22]
LOAD	Y [20]	Not Tested	N (against PVF in Labradors with elbow OA) [20]	Y [20]	Y [20]	Y [20]

Y = Yes, N = No, PFA = Principal Factor Analysis, QOL = Quality of Life, PVF = Peak Vertical Force, OA = Osteoarthritis.

Our primary objective was to test LOAD against other similar measures to provide evidence of construct validity, and against the results of force platform analysis to provide evidence of criterion validity. Secondarily, we wished to report factor analysis of LOAD, to repeat factor analysis of CBPI and HCPI, to repeat internal consistency testing for LOAD, CBPI and HCPI, and to test CBPI and HCPI for criterion validity. For dogs for which we had longitudinal data, we also wished to compare changes in instrument scores with changes in activity data, as collected using accelerometer-based monitors.

Our hypotheses were that there would be correlation between the three CMIs, and that there would be correlation between the CMIs and the objective measures.

## Materials and Methods

The study protocol was approved by the University of Liverpool Research Ethics Committee. A public awareness campaign was run targeting the hinterlands of the University of Liverpool Small Animal Teaching Hospital (SATH) in the Northwest of England, a region with a human population in the region of five million. This included press advertisements and editorials, local radio coverage, poster campaigns, and a letter-drop to previous clients of the SATH’s orthopedic service. Interested pet owners then contacted study personnel and completed a preliminary telephone screening interview. Primary care veterinarians of potential participants were then contacted in writing to request referral for inclusion in the study, in a process approved by the Professional Conduct Department of the Royal College of Veterinary Surgeons. Prospective participants then attended the SATH for a screening visit.

At the screening visit, clinical history was collected and a clinical examination was completed by a veterinarian with experience in orthopedics, and recorded in a standardized data-capture form (DCF). For this study, data from two cohorts, one cross sectional and one longitudinal, were used. The longitudinal cohort is a sub-population of the cross-sectional cohort. Inclusion criteria for the cross-sectional cohort were different than those for the longitudinal cohort. Both are summarized in table 2. Inclusion criteria for the longitudinal cohort were defined by research activity other than that currently reported.

**Table 2:** Inclusion criteria for the cross sectional and longitudinal cohorts.

Cross sectional	Longitudinal
>1 year of age	>1 year of age
>10 kg body weight	>10 kg body weight
Clinical evidence of OA of at least one shoulder, elbow, carpus, hip, stifleor tarsus.	Clinical and radiographic evidence of OA of at least one elbow, hip or stifle.
No other orthopedic disease	No other orthopedic disease
No other disease that may affect mobility, activity or quality of life	No other disease that may affect mobility, activity or quality of life
CMI and FP data captured at the same time-point.	Serum biochemistry (routine profile) and hematology parameters within predefined acceptable ranges.
	CMI, AM and force-platform data for “baseline” and “on-treatment” time points.
	Symmetry index >6.

OA = Osteoarthritis, CMI = Clinical Metrology Instrument, FP = Force Platform, AM = Activity Monitor.

Owners received the instructions, as published for each CMI, before being given ample time in a quiet space to complete the CMIs. For the longitudinal cohort, the same person completed the CMIs at each subsequent assessment.

Force-platform analysis was performed in a dedicated canine gait analysis laboratory. This consisted of a force-platform (Kistler, Switzerland) set in a low-level runway constructed of hard foam. The force platform and runway were covered with the same non-slip surface. Four high-speed, infra-red motion capture cameras (Proreflex, Qualisys, Sweden) were arranged in an arc around the force platform, creating a calibrated motion capture volume approximately four meters long with the force platform at the center. A digital video camera (Sony Corporation, Japan) was directed at the force platform to record each trial for validation purposes. At each session, dogs were allowed a minimum of five minutes to familiarize with the space, and several “practice” trials were performed. Reflective markers were placed bilaterally at the dorsoventral midpoint of the tenth rib of each dog to facilitate velocity measurement of each trial. For each trial, four seconds of force data, motion capture data, and digital video footage were collected simultaneously. Video footage was examined to confirm satisfactory foot placement and motion data were used to measure forward velocity and acceleration: these were both performed using proprietary software (Qualisys Track Manager, Qualisys, Sweden). Force data were analyzed using dedicated software (Bioware, Kistler, USA). Dogs were allowed to move at the gait and velocity that was most comfortable for them and which allowed for most consistent foot placement on the force platform. Once this gait and velocity were identified, it was recorded and kept constant for every trial and, for dogs in the longitudinal cohort, for all subsequent sessions.

For dogs recruited to the longitudinal study, an accelerometer-based activity monitor (AM) (Actical, Philips Respironics, The Netherlands) was attached at the ventral aspect of the collar using nylon cable ties at the end of the screening visit.

For each longitudinal-cohort participant, the study began with a fourteen-day “baseline period”. During this time, no NSAID medication was administered. Owners were provided with a supply of veterinary-licensed paracetamol/codeine tablets (Pardale V, Dechra Animal Health, UK) to use as “rescue analgesia” if they felt necessary. After this baseline period, participants attended “Visit 1″. At this visit, and all subsequent visits, CMI and force platform data were collected as described above, and activity data were downloaded from the AM. Data collected at this visit were used as the ‘off-treatment’, or baseline, data. At Visit 1, dogs were randomly allocated to receive one of two NSAIDs, both licensed in Europe for the long-term treatment of canine OA. Dogs received the allocated NSAID for the next 12 weeks, administered on-label. Data were collected after six weeks of treatment at “Visit 2″, and at the end of the treatment period at “Visit 3”.

Criterion validity was tested in two ways. Primarily, CMI scores were compared against the left-right symmetry index (SI) for the worst affected limb. Symmetry index for PVF was calculated thus:
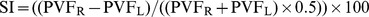
where PVF_R_ is the PVF for the right limb and PVF_L_ is the PVF for the left limb. If the index joint was an elbow, for example, then the SI for the thoracic limbs was calculated. If the index joint was a hip or stifle (knee), the SI for the pelvic limbs was calculated. Negative values (i.e. produced for right limb lameness) were made positive. Total CMI scores were compared against SI. Further to this, following factor analysis, a LOAD “lameness index” was generated and also compared against SI. Secondarily, criterion validity was tested in the longitudinal cohort by comparing change in CMI scores against change in PVF for the index limb, and against change in activity parameters from the AMs, from Visit 1 (baseline) to Visit 2 (six weeks of treatment). Activity parameters used were total weekly count (TWC), and weekly average counts for four quarters of the day: Q1 = 12 am to 6 am, Q2 = 6 am to 12 pm, Q3 = 12 pm to 6 pm, and Q4 = 6 pm to 12 am.

Construct validity was tested, primarily, by comparing LOAD, CBPI and HCPI scores against each other. Additionally, factor analysis was performed for all CMIs and reported for the first time for LOAD, and was compared against that previously reported for CBPI and HCPI. For factor analysis, data from the cross-sectional cohort was used and a Kaiser-Meyer-Olin measure of sampling adequacy >0.6 was used as an indicator for sampling adequacy. Extracted factors were assessed by Eigenvalue, scree-plot analysis and theoretical interpretability. Item loading on extracted components was based on a varimax-rotated model of the factor analysis, with a communality cut-off value of 0.4.

For all comparisons, Spearman’s rank correlation was used and significance was set at p≤0.05 (two-tailed). Internal consistency for all CMIs was tested for the cross-sectional cohort, using Cronbach’sα.

It should be noted that CBPI is reported as a three factor CMI, made up of a Pain Severity Score (CBPI PSS), Pain Interference Score (CBPI PIS), and an Overall Quality of Life Score (CBPI QOL). For all analyses except factor analysis, each CBPI factor was tested individually.

## Results

525 enquiries from interested dog owners were received. 508 telephone interviews were performed, resulting in 362 screening examinations. Of these, 222 dogs met the inclusion criteria for the cross-sectional cohort. Of these dogs, longitudinal data were available for 79 that fulfilled the inclusion criteria for the longitudinal cohort. Demographic data of the cross-sectional cohort are summarized in table 3.

**Table 3:** Demographic data of the cross-sectional cohort.

Variable			Number
Gender (number)	Male	38	222
	Neutered Male	81	
	Female	16	
	Neutered Female	87	
Age (Years)	Mean (SEM)	8.34 (0.21)	220
	Minimum	1.72	
	Maximum	15.02	
Bodyweight (kg)	Mean (SEM)	31.40 (0.85)	221
	Minimum	10.9	
	Maximum	86.0	
Breed (number)	Number of breeds represented	44	222
	Labrador	59	
	Cross Breed	54	
	Border Collie	17	
	German Shepherd Dog	11	
	Golden Retriever	10	
	English SpringerSpaniel	8	
	Staffordshire BullTerrier	7	
	Other	56	
Joint Affected(number)	Elbow	93	222
	Stifle	54	
	Hip	53	
	Carpus	5	
	Tarsus	3	
	Shoulder	1	
	Combination	13	

SEM = Standard Error of the Mean.

For Joint affected, a classification of “Combination” was entered where it was not possible to assign a “worst affected” joint on the basis of clinical history, examination and force platform analysis. Dogs classified otherwise may also have had multi-centric OA, but one joint, or pair of joints, was considered to be the most clinically significant.

**Figure 1:**
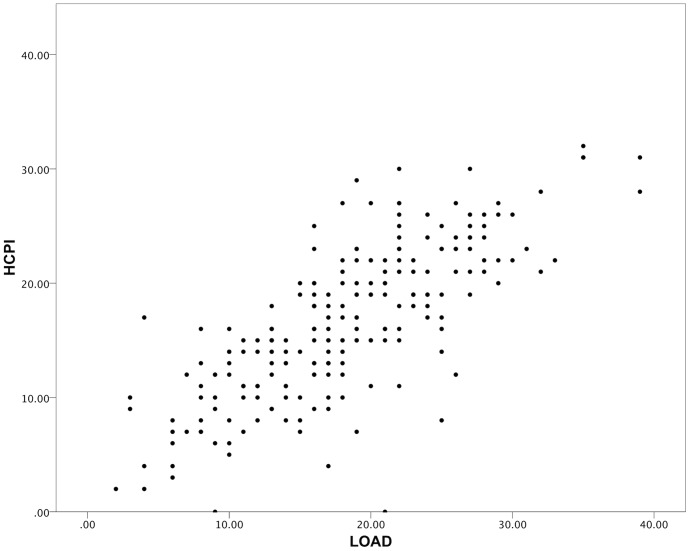
Scatterplot of LOAD versus HCPI scores for the cross-sectional cohort. There is a significant, moderate correlation typical of inter-instrument comparisons. Spearman’s Rank Correlation Coefficient = 0.77 (p<0.01).

**Figure 2:**
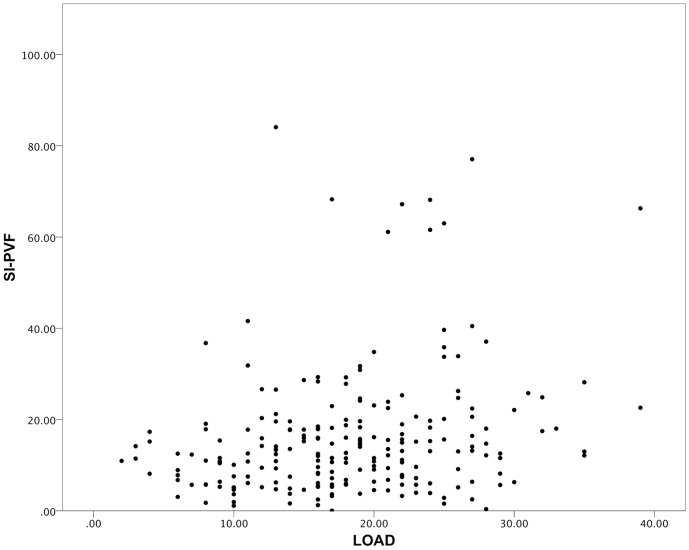
Scatterplot of LOAD versus SI-PVF scores for the cross-sectional cohort. There is a significant, weak correlation. Spearman’s Rank Correlation Coefficient = 0.23 (p<0.01).

Table 4 summarizes the correlations for the cross sectional cohort. Significant moderate correlations were found between all CMIs; and significant weak correlations between SI-PVF and LOAD (r_s_ = 0.232, p<0.01), CBPI PSS (r_s_ = 0.281, p<0.01), and CBPI PIS (r_s_ = 0.276, p<0.01). Figures 1 and 2 are scatterplots illustrating the correlations between LOAD and HCPI, and between LOAD and SI-PVF respectively. In addition to the data tabulated, the LOAD “lameness index” correlated with SI (r_s_ = 0.297, p<0.01, n = 224). Table 5 summarizes correlations between changes in outcome measures for the longitudinal cohort. For the longitudinal cohort, no significant correlations were found between changes in any CMI scores and changes in PVF or any activity parameter. Cronbach’s α for LOAD, HCPI, CBPI PSS and CBPI PIS were 0.88, 0.83, 0.92 and 0.92 respectively.

**Table 4:** Correlations between LOAD, HCPI, CBPI (PSS, PIS and QOL) and SI-PVF for the cross-sectional cohort.

Measure		SI-PVF	LOAD	HCPI	CBPI PSS	CBPIPIS	CBPI QOL
SI-PVF	r_s_	1.00	0.232	0.128	0.281	0.276	−0.196
	Sig.		<0.01	0.058	<0.01	<0.01	<0.01
	n	232	222	221	221	220	221
LOAD	r_s_	0.232	1.000	0.766	0.673	0.795	−0.618
	Sig.	<0.01		<0.01	<0.01	<0.01	<0.01
	n	222	222	220	220	220	220
HCPI	r_s_	0.128	0.766	1.000	0.611	0.738	−0.511
	Sig.	0.058	<0.01		<0.01	<0.01	<0.01
	n	221	220	221	218	218	219
CBPI PSS	r_s_	0.281	0.673	0.611	1.000	0.809	−0.510
	Sig.	<0.01	<0.01	<0.01		<0.01	<0.01
	n	221	220	218	221	219	219
CBPI PIS	r_s_	0.276	0.795	0.738	0.809	1.000	−0.596
	Sig	<0.01	<0.01	<0.01	<0.01		<0.01
	n	220	220	218	219	220	220

r_s_ = Spearman’s Rank Correlation Coefficient, n = sample size for correlation.

Significance is set at p≤0.05 (2-tailed).

**Table 5:** Correlations between changes in LOAD, HCPI, CBPI (PSS, PIS and QOL and PVF and activity parameters for the longitudinal cohort.

Measure		LOAD	HCPI	CBPI PSS	CBPI PIS	CBPI QOL
LOAD	r_s_	1.000	0.735	0.495	0.524	−0.454
	Sig.		<0.01	<0.01	<0.01	<0.01
	n	80	80	80	80	80
HCPI	r_s_	0.735	1.000	0.462	0.561	−0.435
	Sig.	<0.01		<0.01	<0.01	<0.01
	n	80	80	80	80	80
CBPI PSS	r_s_	0.495	0.462	1.000	0.608	−0.366
	Sig.	<0.01	<0.01		<0.01	<0.01
	n	80	80	80	80	80
CBPI PIS	r_s_	0.524	0.561	0.608	1.000	−0.268
	Sig.	<0.01	<0.01	<0.01		0.016
	n	80	80	80	80	80
PVF	r_s_	−0.146	−0.174	−0.168	−0.116	0.199
	Sig.	0.199	0.125	0.139	0.310	0.078
	n	79	79	79	79	79
TWC	r_s_	−0.062	0.034	−0.004	0.056	0.030
	Sig.	0.611	0.783	0.972	0.646	0.804
	n	69	69	69	69	69
Q1A	r_s_	−0.047	−0.002	0.006	−0.140	0.000
	Sig.	0.704	0.987	0.960	0.250	1.000
	n	69	69	69	69	69
Q2A	r_s_	−0.208	−0.085	−0.123	−0.042	0.008
	Sig.	0.086	0.485	0.314	0.733	0.951
	n	69	69	69	69	69
Q3A	r_s_	−0.065	−0.021	−0.084	−0.002	0.095
	Sig.	0.596	0.866	0.494	0.985	0.438
	n	69	69	69	69	69
Q4A	r_s_	0.031	0.017	0.228	−0.014	−0.032
	Sig.	0.798	0.891	0.059	0.912	0.794
	n	69	69	69	69	69

r_s_ = Spearman’s Rank Correlation Coefficient, n = sample size for correlation.

Significance is set at p≤0.05 (2-tailed).

**Figure 3:**
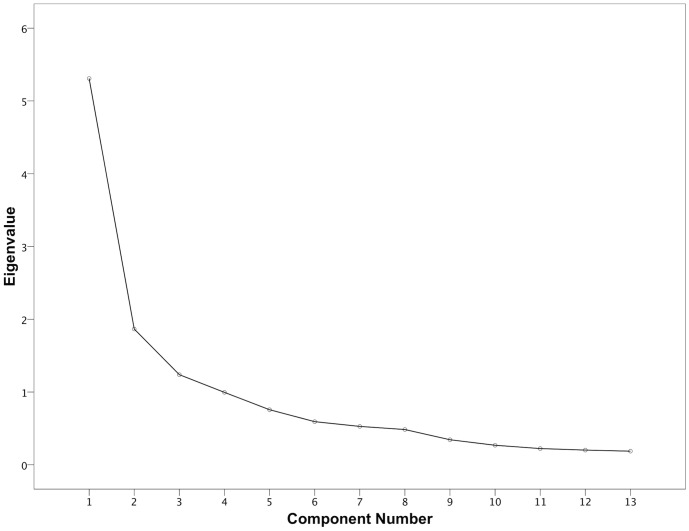
Scree plot of the factor analysis of LOAD. There are three factors with Eigenvalues>1, and no discernible “shoulder” to the plot.

**Figure 4:**
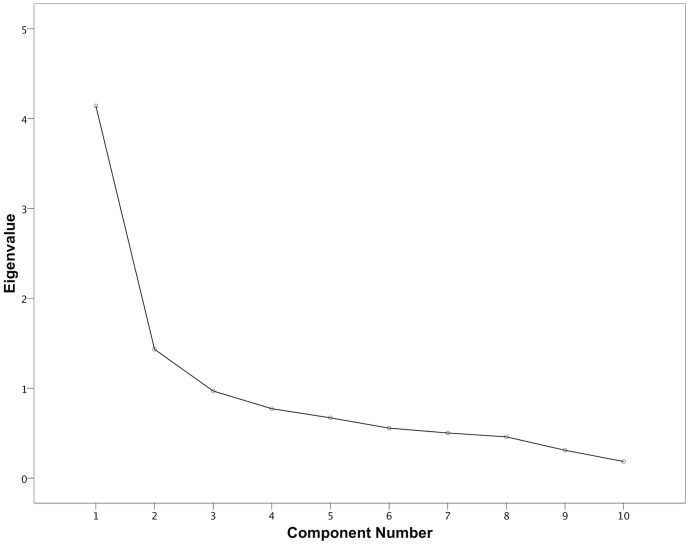
Scree plot of the factor analysis of HCPI. There are two factors with Eigenvalues>1, one factor with an eigenvalue close to 1, and no discernible “shoulder” to the plot.

**Figure 5:**
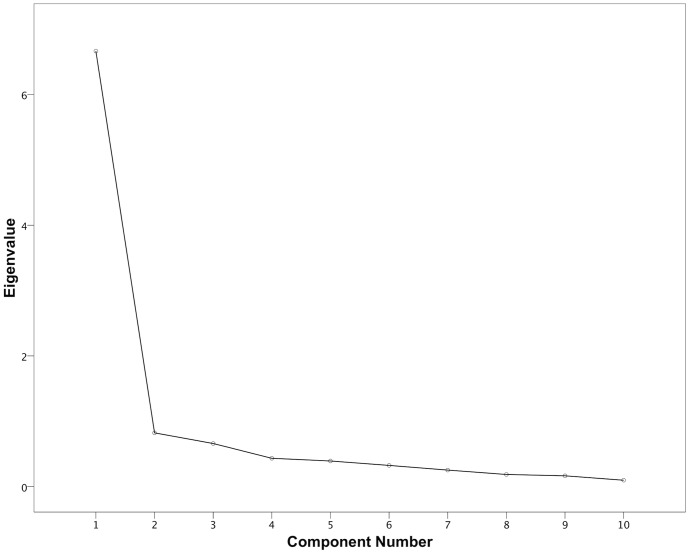
Scree plot of factor analysis of CBPI. There is one factor with an Eigenvalue>1, and a clear “shoulder” to the plot with a single factor to the left of this shoulder.

Kaiser-Meyer-Olin values for factor analyses of LOAD, HCPI and CBPI were 0.83, 0.81 and 0.90 respectively, indicating suitability of data for factor analysis. Figures 3, 4 and 5 depict scree plots for LOAD, HCPI and CBPI respectively. For LOAD, three components with eigenvalues >1 were extracted. These components accounted for 40%, 14% and 10% of the total variance of LOAD respectively. Table 6 summarizes the loading for items on each of the three extracted factors, based on the varimax-rotated solution. For HCPI, two factors with eigenvalues >1 were extracted, and a third with an eigenvalue of 0.99. These components accounted for 41%, 14% and 10% of the total variance respectively. Table 7 summarizes the loading for items on each of these three factors, based on the varimax-rotated solution. For CBPI, one factor with an eigenvalue >1 was extracted. All items loaded heavily on this component with communalities ranging from 0.73 to 0.91.

**Table 6:** Item loading for components extracted by factor analysis of LOAD, based on varimax rotated solution.

Factor	Item/topic	Factor loading	Communality
**1**	**6**. Activity level at exercise	0.88	0.77
	**3:** General activity levels	0.81	0.67
	**8:** Ability to exercise	0.81	0.78
	**7:** Keenness to exercise	0.67	0.51
	**10:** Frequency of rest during exercise	0.55	0.42
	**1:** General demeanor	0.43	0.24
**2**	**12:** Stiffness after exercise then rest	0.82	0.74
	**9:** Effect of exercise on lameness	0.81	0.67
	**5:** Stiffness after rest	0.69	0.59
	**2:** Disability caused by lameness	0.69	0.61
	**13:** Effect of lameness on ability to exercise	0.66	0.73
**3**	**4:** Effect of weather on lameness	0.90	0.87
	**11:** Effect of weather on ability to exercise	0.84	0.82

**“Factor loading”** is the correlation between the item and factor. Loading values>0.4 indicate good correlation of the item with the factor [31].

**“Communality”** represents the proportion of the variance for the item that is explained by the factor. A communality value <0.40 may indicate that the item is not related to the other items in that factor [31].

**Table 7:** Item loading for components extracted by factor analysis of HCPI, based on varimax rotated solution.

Factor	Item/topic	Factor loading	Communality
**1**	**5:** Willingness to trot	0.84	0.75
	**6:** Willingness to gallop	0.77	0.68
	**2:** Willingness to play	0.75	0.61
	**4:** Willingness to walk	0.75	0.63
	**1:** Dog’s mood	0.63	0.41
**2**	**9:** Ease in rising	0.86	0.78
	**8:** Ease in lying down	0.74	0.59
	**7:** Willingness to jump	0.49	0.52
	**11:** Difficulty in movement after exercise	0.40	0.49
**3**	**3:** Audible complaining	0.83	0.71

## Discussion

Here, for the first time, we report evidence of criterion validity for LOAD and CBPI. Criterion validity for LOAD was previously investigated, but not demonstrated, in a small cohort of Labrador retrievers [20]. In that study, LOAD scores for 20 dogs with chronic elbow OA were compared with PVF measured at the same visit but no significant correlation was found. Authors of that study suggested that the multi-dimensionality of LOAD might mean that it is capturing information other than an estimate of PVF. It is also possible that the lack of correlation was a type II statistical error as a result of the small cohort. There is no prior reported testing of CBPI for criterion validity. Criterion validity of HCPI was previously argued on the basis of a correlation between HCPI and a ‘quality of life’ score on a visual analogue scale. However, it is debatable whether a single-item, subjective grading constitutes a ‘gold standard’ or external measure of disease.

The correlations between LOAD and SI PVF and the CBPI factors and SI PVF were significant but weak. However, left-right asymmetry constitutes only one aspect of impaired limb function. Dogs with bilateral disease, for instance, may have severely impaired function but be relatively symmetrical with respect to load bearing. Diametrically, a dog with severe OA in a single joint but three other healthy limbs might have reasonable mobility overall, but marked asymmetry. Likewise, impaired limb function is only part of the construct that these CMIs aim to capture. Therefore, it is not surprising that correlations are only weak.

We chose this symmetry index as our primary criterion variable as it is applicable across all sizes and shapes of dog, across different gaits and velocities, and for thoracic and pelvic limbs. To use simply PVF would mean sub-dividing the cohort by index joint, and normalizing at least to bodyweight, but also arguably to other biometric values. Asymmetry of ground reaction forces has previously been used as a criterion reference for CMI validity testing [21]. We also allowed variation between dogs for gait and velocity. The population was heterogeneous in terms of size and breed, and all data were collected using the same force platform. This meant, for example, that individual foot placement on the platform could only be achieved for small dogs when trotting. Other dogs were so severely impaired that they could not trot for long enough to gather multiple trials, so data were collected at a walk. Similarly, the preferred velocity varied between dogs with body size and degree of impairment. Within each dog, during and between sessions, gait and velocity were kept constant for every trial.

We also tested for criterion validity by comparing change in CMI scores with change in PVF for the index limb, and with changes in activity as measured using an accelerometer-based AM. No significant correlations were found in these analyses. There were, however, statistically significant correlations between changes in all CMIs. The longitudinal cohort was smaller than the cross-sectional one, and it is possible that the lack of significant correlations is due to insufficient statistical power. It is also possible that NSAID treatment affects different components of the OA construct to different degrees, possibly even differently across individual dogs. Change in PVF will only detect a treatment effect on load-bearing through an individual limb: it may not detect an overall improvement in mobility, and would not detect a change in demeanor or activity. Furthermore, the use of AMs as an outcome measure for canine OA remains exploratory. We decided to use TWC and weekly averages for daily quartiles as our activity parameters as these have been previously reported [3], [10], [24] However, it is possible that these parameters are not the most reliable to extract in order to measure the severity of the impact of OA. Part of the reason for the lack of significant association between CMI instrument data and the change in objective measures of limb use and activity may be learned behaviors. Following successful total hip arthroplasty, humans can take many months to ‘unlearn’ what appears to be a learned gait adaptation [25]. The dogs in this study had suffered OA-associated pain chronically, and it is possible that learned gait abnormalities or learned changes in activity affected the sensitivity of parameters in terms of response to the NSAID. Conversely, the CMIs may have detected a positive response to the NSAID by the fact they measure aspects such as demeanor.

Construct validity was tested by comparing CMI scores against each other. For the cross-sectional cohort, significant moderate correlations were found between all instruments. These CMIs have been developed in similar ways, but by different authors in different geographical territories. Item selection and reduction has been based on preliminary testing that has occurred on different cohorts of dogs, belonging to owners from different cultural backgrounds. Furthermore, the scaling of items is similar for HCPI and LOAD (both being based on 5-point Likert scales) but is different for CBPI, which is based on 11-point numerical rating scales. It is likely that each CMI captures different components of the OA construct to variable degrees, therefore correlations are only moderate, and not strong.

Construct validity was also explored using factor analysis. Factor analysis is similar to principal component analysis and is performed in the same way. One difference between the two is that factor analysis assumes that there are underlying factors (latent variables) that have causal influence on the observed variables. The aim of factor analysis in construct validation is not necessarily to reduce the data to simplified components for further analysis, but to determine if extracted components can be reasonably explained by the theoretical construct underlying the instrument. Component extraction based on factor analysis is somewhat subjective, although several recommendations are reported. One such recommendation is to extract only components with an Eigenvalue greater than or equal to one [26]. However, this may be arbitrary and important components may be missed. Another technique, the scree test [27], is to perform a scree-plot of the Eigenvalues and identify the “shoulder” between the steep portion of the curve and the flat part. However, the usefulness of this technique is also limited, because not all scree-plots will have an obvious shoulder. Ready interpretibility of extracted components is an important, subjective assessment: if the items that load heavily on a component seem related, then it is likely that extraction of that component is relevant [28].

Factor analysis of LOAD data extracted 3 components with Eigenvalues >1. Based on the items loading on these components, they could reasonably be identified as 1) an “activity/exercise” component, 2) a “stiffness/lameness” component and 3) an “effect of weather” component.

Factor analysis of HCPI data extracted 2 components with eigenvalues >1, but a third, with an eigenvalue of 0.99 was also evaluated. Based on the items loading on these components, they could reasonably be identified as 1) a “willingness to be active/exercise” component, 2) a “stiffness/ease of movement” component and 3) a “painful vocalization” component. These results are very similar to those previously reported [23]. Interestingly, the first two components extracted for both LOAD and HCPI have descriptive similarities, and both account for similar amounts of variance in the aggregate scores. Conversely, factor analysis of CBPI data extracted only 1 component with an Eigenvalue >1, on which all items loaded heavily. Items within the Pain Interference factor of the CBPI pertain to similar observations as those tested in LOAD and HCPI, for example general demeanor and ability to rise from lying: however, this did not result in extraction of multiple components. This differs from previously reported factor analysis of CBPI [29], which extracted two factors with item loadings consistent with the theorized components of “pain severity” and “pain interference”.

Overall, the data presented here demonstrate significant overlap in the theoretical constructs that underpin these three CMIs, and that the components extracted by factor analysis can be reasonably explained by theorized components of the constructs. This is evidence that the constructs on which these CMIs are based are reasonable approximations of what constitutes canine OA in the clinical sense.

Correlation of LOAD and CBPI with SI-PVF is the first reported evidence of criterion validity for these CMIs. It is not certain why HCPI did not correlate with SI-PVF, but it may related to the observation that factor analysis did not extract a component that could reasonably described as “lameness” based on the wording of the loading items. CBPI PSS and CBPI PIS had the highest correlation coefficients, and it is reasonable to assume that “pain” as captured by this instrument might correlate with lameness. However, the LOAD “lameness index”, as identified by factor analysis, had a higher correlation coefficient still.

Based on these analyses, and those previously published, LOAD can be recommended as a valid measurement tool for canine OA.
